# Fitting the HIV Epidemic in Zambia: A Two-Sex Micro-Simulation Model

**DOI:** 10.1371/journal.pone.0005439

**Published:** 2009-05-05

**Authors:** Pauline M. Leclerc, Alan P. Matthews, Michel L. Garenne

**Affiliations:** 1 Institut Pasteur, Unité d'Epidémiologie des Maladies Emergentes, Paris, France; 2 School of Physics, University of Kwazulu-Natal, Durban, South Africa; 3 Institut pour la Recherche et le Développement, Paris, France; McGill University Health Center, Montreal Chest Institute, Canada

## Abstract

**Background:**

In describing and understanding how the HIV epidemic spreads in African countries, previous studies have not taken into account the detailed periods at risk. This study is based on a micro-simulation model (individual-based) of the spread of the HIV epidemic in the population of Zambia, where women tend to marry early and where divorces are not frequent. The main target of the model was to fit the HIV seroprevalence profiles by age and sex observed at the Demographic and Health Survey conducted in 2001.

**Methods and Findings:**

A two-sex micro-simulation model of HIV transmission was developed. Particular attention was paid to precise age-specific estimates of exposure to risk through the modelling of the formation and dissolution of relationships: marriage (stable union), casual partnership, and commercial sex. HIV transmission was exclusively heterosexual for adults or vertical (mother-to-child) for children. Three stages of HIV infection were taken into account. All parameters were derived from empirical population-based data. Results show that basic parameters could not explain the dynamics of the HIV epidemic in Zambia. In order to fit the age and sex patterns, several assumptions were made: differential susceptibility of young women to HIV infection, differential susceptibility or larger number of encounters for male clients of commercial sex workers, and higher transmission rate. The model allowed to quantify the role of each type of relationship in HIV transmission, the proportion of infections occurring at each stage of disease progression, and the net reproduction rate of the epidemic (*R*_0_ = 1.95).

**Conclusions:**

The simulation model reproduced the dynamics of the HIV epidemic in Zambia, and fitted the age and sex pattern of HIV seroprevalence in 2001. The same model could be used to measure the effect of changing behaviour in the future.

## Introduction

The dynamics of HIV epidemics in Africa remain poorly understood, and virtually no mathematical model has been able to reproduce them accurately. By the year 2000, after some 20 years of transmission of the virus, some countries had high or very high levels of HIV seroprevalence, while others remained with low or very low levels [1]. For a long time the evidence showing the differences between countries remained weak and based on biased and erratic data on HIV seroprevalence among pregnant women. With the development of HIV testing in the Demographic and Health Surveys [2] (DHS) and other large-scale seroprevalence surveys conducted on representative samples of adult populations, major differences in seroprevalence emerged clearly, ranging for instance from 0.7% (Senegal, 2005) to 25.9% (Swaziland, 2006).

Despite these large differences in levels, some features seem to be common to the African epidemics: similar age profiles for adults, and similar differences between men and women. Typically, the HIV seroprevalence is very low before sexual debut, which occurs around age 11 years on the average, rises quickly with age, up to a peak in the 30's, then declines less rapidly with age, the last age available being usually 49 years for women and 59 years for men in DHS surveys. For women the rise of seroprevalence by age is sharper than for men, the peak is around 32 years (range among 21 countries: 27–36), the maximum seroprevalence is about 25% higher than for men (range 0% to 72%), and the decline with age somewhat faster. For men, the rise is slower; the peak is around age 37 (range 34–41), the maximum lower or equal, and the right tail longer than for women. As a result, the lifetime risk of infection is quite similar for both sexes, and women tend to be infected earlier.

The gap between the age at peak infection of men and women is similar whatever the level of seroprevalence, with an average of 6 years for the surveys available (range 3 to 9). These common features of African epidemics are due to the same dominant mode of HIV transmission for adults: unprotected heterosexual contact [1], [3]. This mode of infection implies *a priori* that equivalent numbers of men and women will be infected in the long run, because of repeated exposure, the age gap being explained by the age differences between sexual partners, within and outside marriage, and by special features of sexual behaviour, in particular commercial sex work.

One way to better understand the common features of the HIV epidemics in Africa is to build a mathematical model able to reproduce the patterns of infection, in particular the age and sex patterns found in demographic surveys. We showed in an earlier paper [4] that this was not possible by using compartmental models, primarily because of the constraints imposed on changing sexual partners. We propose here a more complex two-sex micro-simulation model, based on detailed age and sex specific individual behaviours. The main target of this model is to fit the detailed age and sex profiles of HIV seroprevalence, and therefore the age gaps between men and women. Our model differs from previous models, which rarely use detailed information by age and sex, nor realistic values of key parameters such as sexual debut, marriage, divorce or commercial sex. Many other models published in the literature are compartmental models and have different targets, such as to account for an overall level of seroprevalence, to account for the effect of various sexual networks, to evaluate the effect of age difference between partners, or to model the population impact of interventions, such as changing number of partners, improving management of other STIs or mass circumcision [5]–[14]. Some of the previous models are closer to our approach. The work by Anderson and colleagues [9], [10] provided detailed age and sex patterns, but tended to ignore marriage, and made very strong simplifications on sexual debut. One of the closest to our model is probably STD-SIM, a micro-simulation model which is population-based, details sexual behaviour, and allows for co-infection with other sexually transmitted diseases [15]. The main differences between our model and STD-SIM are the more detailed parameterisation of the risk periods, in particular sexual debut, marriage, and divorces, and the mode of partnership formation. On the other hand we ignored the dynamics of co-infection with other STI's, and our aim was focused only on one country. Also very close is the recent micro-simulation model developed at the University of Pau, France, which attempts to fit the HIV situation in Cameroon [16]. The main difference with this new model is the emphasis on commercial sex and the lack of precise reference to marriage. However, the Kamla & Artzouni model is more sophisticated in the transmission module, because it includes detailed dynamics of the viral load during infection, and relates transmission to viral load.

Our model is applied to the case of Zambia. There were several reasons for choosing this country. The HIV/AIDS epidemic was early, large, and well documented: the HIV prevalence increased steadily since 1980, reaching nearly 15% in 2001 [17]. Zambia was one of the first countries to conduct a detailed DHS in 2001 [17], which included age and sex profiles of HIV prevalence, as well as most of the variables needed to build the model. Zambia has also a wealth of detailed and reliable demographic and epidemiologic data, which can be used for the modelling exercise.

## Materials and Methods

### Micro-simulation model

The model is a stochastic discrete-event process with a time-step driven approach, typical of a Markovian process. The model is structured as three main modules. The “demographic module” includes simple renewal processes: births, ageing and deaths; its time step is the month. The “marital module” includes the marriage market (entry and exit from marriage) and the other types of relationships (within marriage, outside of marriage and commercial sex); its time step is the week. Sexual behaviour is modelled within each type of relationship, and its time step is also the week. The “epidemiological module” includes HIV/AIDS infections, either by heterosexual transmission among couples or by vertical transmission from an HIV-positive mother to her newborn child; its time-step is one week for adult transmission, and associated with sexual behaviour, whereas the time-step is one month for vertical transmission, and associated with births. Disease progression after primo-infection is included in this module, up to death. The simulations start from a baseline in 1980, when a seed of HIV is introduced, and run for 25 years, the main target being to fit the situation in 2001. The model is written in C++, and the code is available from the authors on request. The essential equations of the model are presented in Appendix S1.

### Demographic module

The demographic module determines the population structure, by age and sex, with a one month time-step for evolution in time. The baseline population is a stable population generated to fit the Zambian population in 1980, with baseline values of age-specific fertility and mortality rates derived from empirical data (DHS surveys). The associated stable population was computed from Lotka's equations and fertility and mortality rates. Fertility and mortality rates for the non-infected population are assumed to remain constant over time. The corresponding total fertility rate (TFR) was 6.12 children per woman, life expectancy was 47.8 years for males and 50.5 years for females (see Appendix S2, Figure S1, S2). The corresponding intrinsic growth rate was 0.029, which is close to the empirical growth rate estimated for Zambia between the 1980 and 2000 censuses (0.028). More details are available in Leclerc, 2009 [18].

### Marital and sexual behaviour module

**Figure 1:**
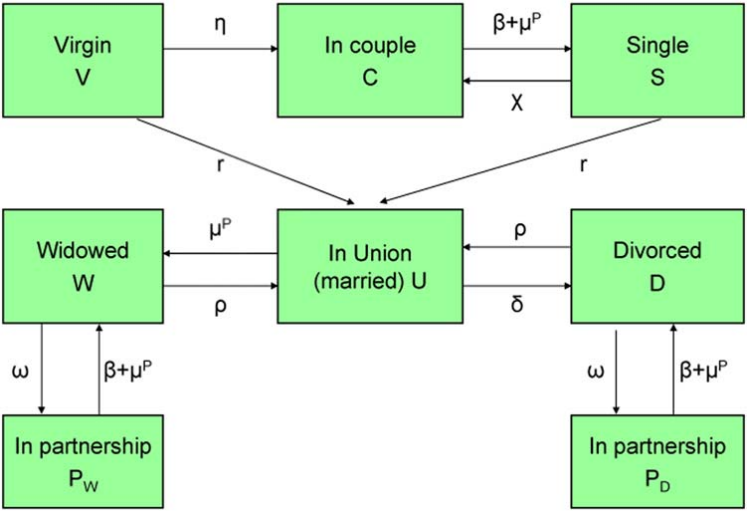
Transition scheme of marital statuses (both sexes).

Three types of relationship were considered, during which HIV heterosexual transmission could occur: marriages (stable unions), casual partnerships (short-term relationships within or outside marriage), and commercial sex. One of the main characteristics of our model is the detailed process of the marital market. At each point in time, each person has a strictly defined status, and during their life course people enter and leave unions, casual partnerships, or commercial sex. There are several possible statuses, with transitions between them (Figure 1): “Virgin” (V) represents people who never had sex; “Single” (S) represents never married people who are sexually experienced; “Couple” (C) represents never married people who are in a casual partnership; “Union” (U) represents people who are married (whether first marriage or remarriage); “Widowed” (W) represents previously-married people who are now widowed; “divorced” (D) represents previously-married people who are now divorced; the two “in partnership” groups (P_W_ and P_D_) represent people who are in a casual partnership and who have been respectively widowed or divorced. Some married men and married women may have casual partnerships, up to three concomitant partners. Some unmarried men and women may also have up to three partners at the same time. Polygyny is also allowed, with up to three wives per husband.

All transitions are random, and controlled by a set of age and sex specific parameters. Individuals enter adolescence as “Virgin” and experience sexual debut, either by first marriage or by couple formation. Transition to first sexual experience follows a parametric model, called the Picrate model [19], fitted with DHS data. The Picrate model is a 3-parameter mathematical function, based on recruitment rates which increase from 0 to a maximum, following a cumulative Weibull function. This parameterization allows one to compute transition rates by week or month. Transitions to first marriage are given by a Picrate model [19], also fitted on DHS data. These functions are displayed in Appendix S2, Figure S3. Casual partnerships end by break-up, at a constant rate. Marriages end by divorce at a constant rate, or by mortality of the partner. Remarriage also occurs at a constant rate, and break as do first marriages. In addition to this main scheme, two types of multiple partnerships were added: polygamy for men, and concomitant marital and extra-marital relationships for both sexes.

Partner selection is achieved through a complex algorithm, designed for fitting both male and female distributions at the same time. In brief, individuals choose partners from the opposite sex in the group of people susceptible to the corresponding status (marriage, casual partnership or commercial sex) depending on an age preference matrix for each type of relation. These preferences are initially given by a bivariate gamma distribution fitted on Zambia DHS-2001 data, specific to the type of relationship, and then fine-tuned by a marital market algorithm in order to balance supply and demand of both sexes. The function is displayed in Appendix S2, Figure S4, S5, S6, S7. Furthermore, the relationship formation rates for previously-married are attenuated at later ages, since empirical data show that the frequency of partnership formation declines with age. For the same reason, the mean number of acts of sexual intercourse was attenuated at older ages of the male partner.

The frequency of intercourse was set at one or two contacts per week per relationship, in order to produce 100 contacts a year for a continuous relationship. This number was independent of the number of concomitant relationships. This assumption reflects the fact that persons who are more sexually mobile (more partners) also tend to have more sex acts per year. The overall number is consistent with the values found in Zambia: 48 contacts per year while taking into account the periods without relationship, and consistent with values found elsewhere, as in the French population [20] and with values used in other models [7], [21]. At each time-step and for each type of relationship, the number of sexual contacts is calculated and applied to the ongoing relationship.

In addition to marriages and casual partnerships, the module allows for commercial sex. Female commercial sex workers are recruited between age 15 and 49, and retire at 50. Women enter the CSW market randomly, selected from the unmarried female 15–49 age group in order to represent, at each point of time, 1% of the unmarried female population aged 15–49 [22]. For males, being a potential CSW client is determined at birth, and some 30% of males are assumed to become clients during their life. This number was derived from an analysis of the 2001 DHS survey [21]. Men who are in this group contact CSW's randomly after their first sexual encounter, and the frequency of contacts depends on their marital status [22]. For more details on this module, see Leclerc et al., 2008 [18].

All the transition rates, constant or age-specific, were calculated beforehand from the 2001 Zambia DHS and are summarised in Table 1.

**Table 1:** Main parameters used in the model: values and sources.

Parameters	Value		Source
*Demographic parameters*	Males	Females	
Total fertility rate (age specific fertility rates)		6.12	DHS Zambia 2001 [17]
Sex ratio at birth	1.00		DHS Zambia 1992, 1996, 2001 [17], [66], [67]
Life expectancy (age specific death rates)	47.8 years	50.5 years	Model life table fitted on DHS data
*Marital parameters*			
Median age at first sex	17 years	16 years	DHS Zambia 2001 [17]
Median age at first marriage	22 years	17 years	DHS Zambia 2001 [17]
Couple formation rate	1.10		DHS Zambia 2001 [17]
Partnership formation rate	1.35		DHS Zambia 2001 [17]
Break-up rate	2.00		DHS Zambia 2001 [17]
Divorce rate	0.015		DHS Zambia 2001 [17]
Remarriage rate	0.254		
Proportion of men with two wives	0.15		DHS Zambia 2001 [17]
Proportion of men with three wives	0.03		DHS Zambia 2001 [17]
Extramarital relation rate	0.19		DHS Zambia 2001 [17]
*Sexual parameters*			
Mean number of intercourses by year	100		[7], [20], [21]
Proportion of CSW's (clients for men)	0.30	0.01	DHS Zambia 2001 [17], [22]
Mean number of visits to CSW per year (unmarried)	4.33*		DHS Zambia 2001 [17], [22]
Mean number of visits to CSW per year (married)	3.03*		DHS Zambia 2001 [17], [22]
*HIV/AIDS parameters*			
MTCT transmission		0.30	Dabis et al. [68]
Fertility reduction		0.30	Hunter et al. [69]
Baseline transmission probability per act	0.0007*		Wawer et al. [24]
Factor HIV stage 1	11.71		Wawer et al. [24]
Factor HIV stage 2 (ref)	1.0		
Factor HIV stage 3	5.0		Wawer et al. [24]
Mean duration of stage 1	0.5 year		Mindel et al. [70]
Mean duration of stage 3	2 years		Mindel et al. [70]

* Theses parameters are allowed to change during simulations.

### Epidemiological module

Heterosexual transmission of HIV occurs in one of the three types of exposure status (marriage, casual partnership or commercial sex). In the case of sero-discordant couples (one partner HIV-positive and the other partner HIV-negative), HIV transmission occurs randomly at each sexual encounter with a given probability. Contamination is therefore simulated by computing the probability of infection given the number of sexual contacts during the at-risk period, that is the duration of the relationship. The basic male to female transmission per act is allowed to change with the stage of the infection of the index case, with age for women, and for the various simulations (see below).

After infection, a person moves through three HIV stages before dying of AIDS: the “primary infection” which lasts 6 months on average, the “latent period” which lasts several years on average, and the final stage “clinical AIDS” which lasts two years on average. Each transition to the next stage, including death, is random, and follows a Weibull distribution defined by the associated median waiting time. Since life expectancy with AIDS decreases with age, the “latent period” is considered to be variable, from more than ten years for people infected at young ages, to about 3 years for people infected after age 50 [23].

All these parameters were derived from the published literature. The duration of primary infection and the stage-specific transmission probabilities were derived from the Rakai study [24]. The age-specific male to female transmission was derived from the Masaka study [25].

Vertical transmission was treated separately. Infected mothers could transmit HIV to children at a constant rate (30%). HIV-infected women had reduced fertility, by a constant ratio (30%).

Survival of the HIV-positive new-born children was calculated independently, and was fitted with a double Weibull distribution, to match the two forms of the disease: rapid-evolution and slower-evolution [26], following the recommendations made by UNAIDS to model child mortality of infected children [27].

### Assumptions about heterosexual transmission of HIV

As will be seen below, the basic parameters described above did not permit the fitting of the empirical data. Therefore, in order to fit the age patterns of prevalence observed in Zambia in 2001, we developed various assumptions concerning the heterosexual transmission of HIV, and in particular: differential susceptibility of young women, and healthy carriage.

Differential susceptibility of young women is based on an observation made in the Masaka study [25]. In sero-discordant couples, the transmission from males to females was higher for women below age 25 than for women above age 25. We built on this observation to test the impact of differential susceptibility by age with our model. The pattern of differential susceptibility by age is presented below.

The second assumption, called “healthy carriage”, was a speculative hypothesis made earlier in order to reconcile the incompatibilities between male and female age profiles of HIV seroprevalence. This hypothesis, developed by MG [28], builds upon the complex mode of infection of the HIV virus, from epithelial cells to blood cells, both processes being highly probabilistic. It assumes that, after exposure to an infected woman, a man could host for a few days the HIV virus in his epithelial cells without being fully infected, therefore remaining seronegative. If such a man had intercourse with a second woman, not infected with HIV, within a short period of time (about one week), he could theoretically transmit the virus to the second woman. This could occur typically in case of concomitant relationships, especially in the case of commercial sex.

### Simulation process

Before starting the simulations, the demographic and marital modules were run several years before the introduction of HIV/AIDS in the population. Then, in 1980, the assumed date of the first HIV cases in Zambia, the virus is introduced in the population, by infecting 1% of the 15–49 age group. The dynamics of the epidemic are then simulated year by year, until 2001. The population characteristics and the infections are monitored over time and stored after each year, so that all details can be retrieved at any point in time.

Several parameters were allowed to change in order to fit the prevalence in 2001. (1) probability of transmission per sex-act; (2) differential susceptibility of women; (3) number of visits to commercial sex workers for unmarried and married men or higher transmission for clients of sex workers; (4) healthy carriage. The simulations explored the realistic combinations of these parameters in their ability to fit the observed patterns in 2001. Above all, we used the flexibility of the HIV transmission probability to fit the level of prevalence in the population, the other parameters being used for fitting the age and sex profiles. For precise fitting of the overall level of seroprevalence, we used Brent's method and the Golden Section search procedure.

Because results of our simulations depended on a large number of parameters, we had to set limits on the values to be taken by varying parameters, called “realistic values”. The heterosexual probability per sex-act in stage 2 was allowed to vary between 0.0007 (value from the Rakai study) and 0.0050; the annual number of visits to commercial sex workers was allowed to vary between 3 and 12; healthy carriage was assumed to vary from 0 to 1, that is the probability to become HIV healthy carrier during one week by contact with an HIV-positive female partner. Different scenarios for differential susceptibility were tested, allowing the female risk of acquiring HIV to be multiplied by a factor between 1 and 5 depending on the age-group.

The empirical age and sex seroprevalence patterns derived from the 2001 DHS survey were affected by random fluctuations. The profiles were therefore fitted with a polynomial on the logit of the seroprevalence: female HIV prevalence peaked at age 31, with 25.7% of infected women, and male prevalence peaked at age 36 with 20.6% of infected men, that is a ratio of female maximal prevalence to male maximal prevalence of 1.25, all values quite typical of African HIV epidemics. These were the main criteria that we used to test the reliability of our simulations, in addition to plotting the corresponding figures.

Finally, most of the events occurring during simulations being stochastic, we used a large sample size (675,000 persons at baseline, or about 12% of the total population) in order to reduce variability in the results of simulations. This variability remained small, especially with this large population size and given the high prevalence levels seen in Zambia.

## Results

Table 1 gives the baseline parameters used in our simulations. With these values, and without adding any of the alternative hypotheses, no epidemic could be generated: the HIV prevalence decreased soon after the introduction of the virus in the population, leading to extinction.

Changing the transmission probability allowed an epidemic, but the final age and sex profiles were far from those expected. With a transmission probability equal to 0.004379, overall prevalence rates at age 15–49 were close to what was expected : 16.5% and 12.8% for female and male groups respectively (as compared with 16.6% and 12.0% respectively in the DHS) but peaks were reached at ages 28 for female and 32 for males, much earlier than expected (31 and 36 years respectively). Moreover, assuming the same value of transmission for males and females, the peak prevalence ratio (F/M) was 1.13, lower than the value in the DHS (1.25).

### Simulation H0

After exploring a wide range of parameters, we found a combination giving a good fit to the age and sex patterns of prevalence in 2001. This simulation is labelled “H0” in this paper, and defined as follows:

- a baseline (stage 2) transmission probability from woman to man of 0.002479;
- a differential susceptibility for young women equal to 1 for women aged more than 40 years, 1.5 times higher for woman aged between 30 and 40 years, and 2.5 times higher for woman aged less than 30 years;
- a four-times higher annual number of visits to commercial sex workers for eligible married men (12 visits), or a four-times higher transmission rate in case of contact with a CSW.

**Figure 2:**
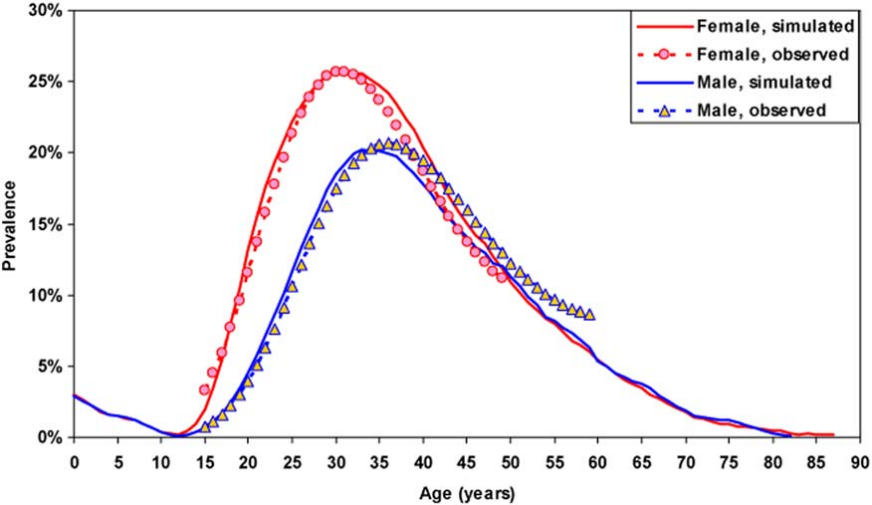
Age patterns of HIV prevalence in 2001 in Zambia, observed (from DHS data) and simulated (from simulation H0) (women in red and men in blue).

The age patterns of prevalence in 2001, simulated and observed, are displayed in Figure 2. The fit is of good quality, even though it was difficult to obtain: the peak prevalence (25.8%) for women occurs at age 31, and the peak prevalence for men (20.5%) occurs at age 35. The peak prevalence ratio (F/M) equals 1.26, and the overall prevalence rates are 17.5% for women aged 15–49 and 11.5% for men aged 15–49 (as compared with 16.6% and 12.0% respectively in the DHS). More important, the age patterns obtained by the simulations were close to those found in the DHS survey. The hypotheses underlying this simulation remained within the range of acceptable values. The transmission probability was 2.5 times that found in discordant couples in Uganda, a value usually considered too low because of a selection bias (couples who have a lower transmission rate are more likely to be discordant). The pattern of differential susceptibility was close to that found in Masaka. However, the annual number of visits to CSW's may seem unrealistic, since it is four times higher than that found in surveys, and therefore assumes a large understatement, but the same results could be obtained with four-times higher transmission rates, which is consistent with the likely presence of co-infection with STI's.

The model allowed us to disentangle the modalities of the transmission, in particular the type of relationship, the age at infection and the stage of the disease at time of infection. For men, a large proportion of infections resulted from contacts with female sex workers (47.2%), followed by contacts during casual partnerships (30.3%), and contacts within marriage (22.5%). For married men, 58.9% of infections resulted from contacts with commercial sex workers, whereas this proportion was only 28.6% for unmarried men. For women, and because they marry early, a majority of infections occurred within marriage (62.5%), followed by casual partnerships (34.9%), commercial sex accounting for a tiny proportion (2.6%), because CSW's account for only 1% of the population. It should be noted however that after 21 years some 90% of CSW's were infected. Moreover, 66.4% of women infected within short-term relationships were infected before their first marriage, which accounts for 22.2% of infections. For men, 75.1% of male infections within short-term relationships occurred before their first marriage, and account for 20.7% of infections.

The difference in age at infection stems from the age differences of partners by type of relationship involved. For infections occurring within marriage, the mean age at infection for men was 36.7 years (IQR = 27 to 45), and for women 27.9 years (IQR = 20 to 33). In contrast, for infections occurring during casual partnerships, ages were younger and the age difference was smaller: 28.1 years for men (IQR = 20 to 33), and 26.6 years for women (IQR = 17 to 33). For commercial sex, the mean age at infection was 33.3 (IQR = 24 to 41) for men, and 26.4 for women. (IQR = 18 to 35). It is therefore primarily the difference in age at marriage that explains the overall age difference at infection.

With respect to the stage of infection, a majority of male infections occurred with a partner in stage 2 (Table 2). Indeed, a majority of infections occurred with a CSW, which explains the large number of infections in stage 2, since CSW's are infected in large numbers and at an early age and therefore are in stage 2 for a large part of their professional lives. In contrast, women get infected mainly by partners in stage 1, because of the high risk associated with this stage during stable relationships. For short-term relationships, female infections occur more often with partners in stage 2, because of the longer duration of this stage.

**Table 2:** Proportion of infections occurring at each stage of the disease in the partner, by type of relationship, and mean age at infection, by sex and type of relationship (H0).

	Women				Men			
	Marriages	Casual partnerships	CSW	Total	Marriages	Casual partnerships	CSW	Total
*Proportions by stage*								
Stage 1	36.9%	10.8%	1.2%	48.9%	3.1%	9.0%	16.1%	28.2%
Stage 2	19.0%	18.1%	0.7%	37.8%	15.9%	16.7%	21.0%	53.6%
Stage 3	6.6%	6.0%	0.7%	13.3%	3.5%	4.6%	10.1%	18.2%
Total	62.5%	34.9%	2.6%	100%	22.5%	30.3%	47.2%	100%
*Mean age at infection by stage*								
Stage 1	29.1	23.0	28.7	27.7	35.2	22.8	33.9	30.5
Stage 2	23.0	23.8	25.0	23.4	34.3	27.4	33.1	31.7
Stage 3	35.1	41.2	23.9	37.2	48.8	40.8	32.9	38.0
Total	27.9	26.6	26.4	27.4	36.7	28.1	33.3	32.5

#### Changing pattern of transmission over time

The proportion of male infections from a CSW varies with the duration since the onset of the epidemic. Before 1985, male infections due to commercial sex account for 73.1% of the total, whereas after 2000 they account for only 36.4%. As many clients of commercial sex workers are married, female infections occurring within marriage also decrease, from 70.0% of female infections before 1985 to 60.3% of female infections after 2000.

#### Net reproduction rate (*R_0_*)

The net reproduction rate (*R_0_*) of the epidemic was calculated by computing the secondary attack rate by year since infection and multiplying by the survivorship of the index cases by year. Results give estimates of *R_0_* equal to 1.95 (2.28 for female to male infections, and 1.61 for male to female infections), which was close to the empirical *R_0_*, defined as the ratios of simulated new infections from 1985 and 2005 to the infections that occurred from 1980 to 1984 (27,650 men and 17,607 women infected from 12,311 women and 11,585 men, or R_0_ = 1.89). This value matches quite well what is known of the dynamics of the HIV infection in Zambia over the period, that is an increase from a low value (about 1%) in 1980 to a high 15% HIV seroprevalence in 2001.

### Other simulations: impact of changing parameters

In this part, we investigate the effect of changing critical parameters around H0: heterosexual transmission probability, differential susceptibility, number of visits to CSW's, and by introducing healthy carriage. Table 3 summarises the various assumptions made with their main results.

**Table 3:** Assumptions made to test the impact of key parameters and their results on key indicators after simulations.

Hypothesis for simulations		Key indicators from simulations						
	(Changes from H0)	Age at peak		Prevalence at peak (%)		Maximal prevalence ratio F∶M	15–49 years-old prevalence (%)	
		Women	Men	Women	Men		Women	Men
DHS	Survey values	31	36	25.7	20.6	1.25	16.6	12.0
H0	Realistic simulation	31	35	25.8	20.5	1.26	17.5	11.5
H1	No differential susceptibility	34	35	18.8	15.6	1.21	11.6	8.4
H1′	H1+Pt = 0.003194	32	34	25.8	21.9	1.18	16.7	12.1
H2	Mean number of visits to CSW for married men = 3.03	28	33	13.8	10.4	1.33	9.4	6.3
H2′	H2+Pt = 0.003279	27	32	26.1	21.9	1.19	17.9	12.6
H3	Healthy carriage	29	34	41.9	33.8	1.24	28.7	18.7
H3′	H4+Pt = 0.002459 with no differential susceptibility	33	35	25.9	21.3	1.22	16.5	11.4

Note: Hn′ are made to fit the overall seroprevalence with other parameters equal to Hn.

Pt = probability of HIV transmission per sex-act.

#### Removing differential susceptibility (H1)

Removing the differential susceptibility of young women induces lower prevalence for both sexes, and higher mean age at infection, especially for women (mean = 29.8 versus 27.4 in the previous simulation). More female infections occur within marriage than previously (71.0% versus 62.5%), and fewer infections occur during short-term relationships (25.1% versus 34.9%). For men, more infections occur while visiting a CSW (59.3% versus 47.2%). Concerning ages at infection, the main difference is observed within casual partnerships. Female mean age at infection within casual partnerships is now 30.7 years (versus 26.6 under H0), because they occur more often after the breaking of the first marriage. In order to fit the levels of prevalence after removing the differential susceptibility for women (H1′), a higher transmission probability by sex-act of 0.003194 is needed (29% higher than H0). With such a transmission probability, female maximal prevalence equals 25.8%, close to what we expected, but male maximal prevalence now equals 21.9% with the result that the ratio of female to male maximal prevalence becomes 1.18 (Table 3), which is lower than what is found in the DHS. Removing differential susceptibility has therefore major shortcomings for the quality of the fit, because too many infections occurred within the male group.

#### Changing the number of visits to CSW for married men (H2)

In this simulation, the mean number of visits to CSW for married men is changed back to its original value (3.03 visits a year). This implies that fewer male infections occur through client-CSW relationships (34.5% versus 47.2% previously) and, as a result, that fewer female infections occur within marriage (54.4% versus 62.5%). Then, because sexual activity for the high risk group of married men clients of CSWs is reduced, fewer infections occur at older ages for men as well as for women. As a result, the mean ages at infection are younger than previously (31.5 years for men, 26.8 years for women), primarily because late infections no longer occur (Table 3). Peaks of prevalence are reached 2 or 3 years before those obtained under H0 (28 years for women and 33 years for men). The main difference in terms of stage of the disease is observed for married women: the proportion occurring in stage 1 falls to 42.2%, whereas the proportion of infections in stage 2 increases to 43.9%. In order to fit the correct levels of prevalence (H2′), the transmission probability by sex-act should be 0.003279 (32% higher than H0). Under this new assumption, HIV prevalence peaks at 27 for women and 32 for men, and the age patterns no longer fit the DHS data.

#### Assumption of healthy carriage (H3)

The assumption of healthy carriage was added this way: all men are susceptible to be a healthy carrier and, for each contact with an HIV-positive partner, they have a probability of 30% to carry the virus during one week without getting infected. After that, if they have contact with other HIV-negative women, during this same week, they could transmit the virus to them with the same transmission probabilities as if they were really infected. The choice of 30% is the result of several simulations and will be explained below.

Adding the healthy carriage hypothesis to H0 implies higher prevalence rates for both sexes with a maximum prevalence equal to 41.9% for female and 33.8% for male (Table 3). The overall prevalence for men and women aged 15–49 were respectively 18.7% and 28.7% in 2001. As women become more susceptible because of healthy carriage, age at maximum prevalence is younger than under H0 (29 years old versus 31). This is the result of a lower mean age at infection for females (26.0 years). As previously, a majority of female infections occur within marriage (63.1%), then within short-term relationships (32.4%) and a small proportion within the commercial sex market (1.6%). Overall, 28.1% of female infections are due to healthy carriage, especially within marriage (35.4%), then within short-term relationships (16.1%). Few female infections attributable to healthy carriage occur within commercial sex (5.6%). For men, the main difference is that they are now as infected in stage 1 (41.9%) as in stage 2 of the disease (45.9%). The proportion of male infections occurring within marriage is twice that under H0 (43.4% versus 22.5% under H0). In summary, men visit commercial sex workers and become healthy carriers; returning home they infect their wives, but get infected soon after that because of the very infective stage 1 of their wives. In order to fit the correct levels of prevalence for both sexes (H3′), we had to remove differential susceptibility. Indeed, it was impossible to fit levels of prevalence for both sexes by combining differential susceptibility and healthy carriage. Under this assumption, we need a transmission probability by sex-act of 0.002459, which is similar to the one used under H0. Prevalence rates peak at ages 33 and 35, and the age patterns no longer fit the DHS data.

## Discussion

To our knowledge, no other model has tried to fit the HIV seroprevalence age patterns for both sexes simultaneously, while taking into account the detailed periods at risk and fitting precisely entry into sexual life, entry into first marriage, and marriage dissolutions as well as re-marriage. To give a simple example, most models assume that all adults enter their sexual life (or first marriage) at exactly age 15, whereas in our model men and women may enter sexual life at any age between 10 and 30, as they do in real life. This is obviously very important to enable proper fitting of the age and sex pattern of infection.

Our simulation exercise aimed at being as realistic as possible, and used as much as possible empirical and detailed age-specific values of the main parameters controlling couple formation and transmission of the virus. Above all, it shows the very heavy constraints for fitting properly the observed data on seroprevalence by age and sex. Changing one parameter has an impact on the whole transmission process, and when it affects directly one sex, it also affects as a consequence the other sex, changing therefore the dynamics of the epidemic and the age and sex profiles. Our reference simulation (H0) was obtained after more than one hundred simulation trials, all the others leading to inconsistent patterns. Even if H0 could be criticised, it has the main advantage of reproducing the pattern observed in the Zambian population and therefore providing a plausible scenario.

Among the main constraints found in the simulation was the age at peak infection for males. It was almost impossible to reach values greater than 35 or 36 years for men while keeping the main parameters within a range of realistic values. This point definitely deserves further research, but this observation seems to match observations in empirical data throughout Africa.

A nice feature of this model is that it disentangles the transmission process. The way the disease is transmitted appears complex, because it involves differently the various types of union formation, and the various stages of transmission. The role of each factor evolves over time, and is sensitive to changing any of the parameters. This is probably why we received conflicting evidence from field surveys conducted over the past 20 years in many African countries. For example, some authors found a correlation between the number of CSWs and HIV prevalence levels across African countries [29], whereas the 4-city study concluded that sex work could not explain the differential spread of HIV among the four cities [30], even though authors acknowledged that it could have played a major role at the onset of the epidemic.

Despite its nice explanatory power, our model has a number of limitations, firstly the values of its parameters. The heterosexual transmission probability is one of the parameters most open to criticism. The Rakai study gives a baseline value of 0.0007 per act [24] (prevalent cases group, stage 2), and an overall transmission parameter of 0.0011 [31] (prevalent and incident cases together), which is about half the values selected for H0. However, our value does not seem too unrealistic, and compares with that selected by other authors [32]–[36].

Differential susceptibility of men and women remains a matter of controversy. Some studies found that women are twice as susceptible as men [25], [32], whereas other studies found no significant difference of transmission between the two sexes [31], [37], [38]. Note that in some studies the differences between male to female and female to male transmission is hampered by male circumcision. It is striking to note that in Europe as in Uganda, where male circumcision is rare, transmission is the same either way. This is why we chose the same value of transmission for males and females for older ages, since circumcision is rare in Zambia. Differential susceptibility data were derived from a study in Uganda. Fortunately, the situation in Zambia is quite similar to the situation in Southern Uganda, with little circumcision, same religion (Christian), and roughly the same level of economic development.

Our assumption of a differential susceptibility by age among women was necessary to fit the observed patterns. It has however an impact on the overall susceptibility of women. Assuming that women have intercourse between age 16 years (median age at first sex) and age 50 years (end of reproductive life), with an average frequency of 80 sex acts per year, and assuming a differential susceptibility as assumed in H0, a simple calculation gives an increased risk for all women of 1.74, which matches other observations in the literature, and the assumptions made in other modelling projects [39], [40].

Differential susceptibility induces more infection at young ages for females and results in a ratio of maximal prevalence (female/male) close to that observed in DHS surveys (1.25) [17]. The assumption of differential susceptibility is supported by studies which found that age might be an important co-factor of HIV infection for women [41], which might be a biological effect. Indeed, the vaginal epithelium of adolescent and young women is thinner than at older ages. In animal models, age was found as a factor of thickness and integrity of the vaginal epithelium [42]. Removing differential susceptibility and adjusting transmission probability leads to a ratio of 1.18. So, discrepancies in gender prevalence are in part explained by the sexual network, but not sufficiently to explain all the differences. To reach such prevalence differences between the two sexes, women have to be more susceptible than men.

Our mean number of partners was derived from DHS data, after a detailed analysis by age and sex. It is somewhat lower than the number used in other models, but we feel that it is realistic for the Zambian situation.

The mean number of sex acts by year for a steady relationship was set to 100 which is a little higher but remained consistent with the values used in other models [7], [21]. We also included a decline of this mean with age, corresponding to a lower sexual activity for older age-groups [43]. Considering a lower number of annual sex acts would simply imply higher transmission per act in our model, but will not change very much the age and sex patterns.

In our simulations, a large proportion of female infections occur within marriage, because of extra-marital relationships of men, including commercial sex. This conclusion is supported by epidemiological [44]–[47] and serological [48] studies. Lurie et al. [44] found that in 71.4% of discordant couples the male was the infected partner and that he was infected mostly outside his regular relationships. Another study concluded that men were four times more likely than women to introduce HIV infection in concordant-negative couples [45]. Moreover, Glynn et al. [49] estimated that at least 25% of the infections in recently married men were acquired from outside the marriage, by extra-marital partnerships. This is mainly explained by gender differences in sexual behaviour, as men more frequently engage in extra-marital relationships, including commercial sex.

Age at first marriage has been shown to be an important factor of HIV prevalence at country level, also demonstrated with its correlate, the prevalence of premarital fertility [50], [51]. Late marriage for women implies long periods of premarital sexual activity during which the rate of partner change can be high, which facilitates the spread of the virus. The case of Zambia is interesting and peculiar because this country has a high level of HIV prevalence despite low median age at first marriage. As a result, most infections occur after first marriage, a situation different from other countries in Southern Africa, where infections mainly occur in the premarital period.

Because the stage of primo-infection is very infectious, women with an infected husband tend to become infected soon after their spouse. In simulation H0, 59.3% of female infections occurring through marriage occur during this 6-month period of primo-infection.

Commercial sex was shown to play a major role in the spread of HIV in the first years of the epidemic in Africa [52]–[55]. In generalized epidemics, however, this role seems less important. The 4-city study conducted in the late 1990's concluded that commercial sex could not explain differential prevalence within the sites [30]. We showed from five case studies in the early 2000s that the role of female sex workers seems limited, and accounts for only 1.3 to 9.4% of infections in the general population, although Zambia was not included in this sample [56]. To a certain extent, results of our simulation H0 reflect this fact. At the onset of the epidemic, the proportion of male infections occurring during contact with CSW's is high (73.1% before 1985), then it decreases to reach 36.4% after year 2000. Moreover, using our first estimation of commercial sex, and after adjustment of the transmission probability (H2′), some 39.8% of male infections are due to commercial sex before 1985, whereas this proportion falls to 21.8% after year 2000. As a matter of fact, the proportion of female infections within marriage decreases from 54.8% before 1985 to 50.2% after 2000. It still remains high compared to our estimate, but reflects, to a certain extent, the fact that the epidemic in Zambia is now generalised to the whole population and not restricted to some high-risk groups.

Our model had several other limits, beyond the values of its parameters. Firstly, heterogeneity in sexual behaviour is represented only by behaviour associated with marital status, and by random effects. There is evidence of more complex heterogeneity, and in particular by more complex “assortative mixing”, that is a preference from both sexes to form sexual partnerships with persons with similar behaviours (either with low or with high number of partners). This is only partly taken into account in our model. Also, we did not take into account preferences for CSW's, that is the fact that some men tend to have relations preferentially with the same person for a long period of time. We also assumed independence between many parameters, such as divorce rates and sexual behaviour, which may differ from real life situations. Some of the parameters were taken as constant, when in reality they are dependent on some other factors. We took a weekly step for the epidemiological module, which implies some constraints in terms of sexual contacts and concomitant partnerships.

We also ignored deliberately other factors of HIV transmission, such as herpes or other STIs, which would require much more complex modelling, and has already been treated elsewhere [8], [57]. The average effect of STI's can be considered to be somehow included in the average transmission rate, and in the excess risk for intercourse with a CSW.

We also ignored the spatial dimension of disease spread, in particular the role of migration, and the differential behaviour in urban and rural areas. These other factors need a separate treatment, and indeed other types of models. The role of the mines, as they attract young unmarried adults and favour commercial sex, is also of concern for Zambia.

We also ignored other routes of transmission, in particular iatrogenic transmission (blood transfusion, unsafe injections or medical practices), sometimes considered to be important [58]. We acknowledge that they may exist, but we thought that they were unlikely to explain the age and sex patterns of HIV infections which was our main target [59].

Our study is primarily heuristic, and aims at explaining a common feature of African epidemics: the age and sex patterns of seroprevalence in the adult population. It may also have some policy implications. By better understanding the likely routes of transmission, one might better target prevention policies. Of course, our findings are country and period specific, and cannot be easily extrapolated to other situations, unless more simulations are run with different parameters. However, they reveal at least two major target groups: sex workers and their clients, and newly married women. If the first group has been the target of many interventions [60]–[63], the second group has largely been ignored as a potentially high risk group [64]. Prevention programs among this group are needed, and this conclusion is supported by epidemiological studies [45].

Our model can also be used for measuring the effect of changing behaviour. After fitting the 2001 situation, one could extrapolate the trends to the next 5 or 10 years. This exercise shows that in 2006, one expects a rising epidemic, with 19.4% of infected women aged 15–49, and 12.4% of men in the same age group; corresponding figures for 2011 are: 20.7% for women and 13.1% for men, with maximal prevalence of 30% for females and 24% for males. On the contrary, seroprevalence seems to have been levelling off and even going down in Zambia over the past years, according to sentinel sites [65]. This tends to indicate that prevention efforts have been successful, and that the course of the epidemic has been curbed by changing behaviour, whether by reducing the number of partners or by using condoms.

Much remains to be explored to better understand the dynamics of HIV epidemics in Africa, and their wide diversity. In particular, may such micro-simulation models help explain the differences in prevalence levels and in age and sex patterns seen over the continent? This remains to be seen by applying the model to other situations.

## Supporting Information

Appendix S1 (0.12 MB DOC)

Appendix S2 (0.32 MB DOC)

Figure S1 Age pattern of fertility, Zambia.
(0.11 MB TIF)

Figure S2 Age pattern of mortality, without HIV/AIDS, Zambia.
(0.12 MB TIF)

Figure S3 Proportions ever married, and ever had intercourse, after from fitting with the Picrate model.
(0.14 MB TIF)

Figure S4 Bivariate gamma distribution of age of husband and wife, first marriage.
(0.21 MB TIF)

Figure S5 Bivariate gamma distribution of age of husband and wife, remarriage.
(0.22 MB TIF)

Figure S6 Bivariate gamma distribution of age of partners, premarital relationship.
(0.20 MB TIF)

Figure S7 Bivariate gamma distribution of age of partners, extra- or post-marital relationship.
(0.22 MB TIF)
