# Supplementary material for: Fitting the HIV Epidemic in Zambia: A Two-Sex Micro-Simulation Model
Source: PLoS One. 2009 May 5;4(5):e5439. doi: 10.1371/journal.pone.0005439 (PMC2673026; doi:10.1371/journal.pone.0005439)
Supplement: Appendix S1 — (0.12 MB DOC) [file pone.0005439.s001.doc]

### Appendix A – Model Equations

# Demographic Module

The equations for the demographic module have a particularly simple form. Births are computed from the probability distribution generated by the age-specific fertility schedule *f*(*a*), non-AIDS deaths from the age-specific non-AIDS mortality schedule *μ_0_*(*a*), and ageing is computed for each individual by adding time-increment δ*a* to age at each time-step.

$$\frac{dN}{da}=-\mu_{0}N_{0}-\mu_{a}N'$$

$$B=\int_{\alpha}^{\beta} N^{F}(a)f(a)da+\int_{\alpha}^{\beta} N'^{F}(a)f'(a)da$$

where:

*N(a,t)* is the population age (a), at time (t), N_0_ the non-infected population

*N’(a,t)* is the HIV infected population age (a) at time (t)

*B(t)* are the births at time (t)

*f(a)* are the age specific fertility rates for non-infected women and f’(a) for HIV+ women.

*µ_0_(a)* is the age specific death rate without AIDS

*µ_a_(a)* is the death rate from HIV/AIDS

# Marital Module

The marital module computes the formation of new marriages (both first marriages and remarriages, including polygamy), casual partnerships, and their dissolution. Commercial sex contacts are also modelled, including recruitment and withdrawal of commercial sex workers, and client-CSW contacts, according to rates specified in the simulation. During the simulation, partners are randomly selected for new unions from lists of susceptible individuals, according to a two-sex probability distribution computed as described below. The formation and dissolution of unions results in transition of individuals between various partnership states, and the evolution of the mean sizes of these states is governed by a set of partnership state equations.

The partnership states are (where *V, S* etc. are proportions of the cohort in that state):

No sexual experience (“virgin”) *V*

Sexually experienced, never married, single *S*

Never married, in a casual partnership *C*

Married (“unions”) *U*

Divorced *D*

Widowed *W*

Previously divorced, in casual partnership *P_D_*

Previously widowed, in casual partnership *P_W_*

The transition rates between states are described in Table A1. The Picrate model for first sex and first marriage recruitment rates, for specific starting age *a*_0_ and maximum recruitment rate *r_max_*, is

$r\left(x\right)=r_{\max}\left(1-e^{-\frac{1}{2}x^{2}}\right)$, where $x=r_{\max}\left(a-a_{0}\right)$. (A1)

The function for late-age attenuation of partnership formation rate and frequency of sexual contacts, is computed at age *a* in a specified age range *a_min_* to *a_max_*, and is given by

, where $u=\frac{kx}{1-x}$, and $x=\frac{a-a_{\min}}{a_{\max}-a_{\min}}$. (A2)

The equations that govern the proportions in each partnership state in a stable population, following an age cohort, are:

$\frac{dV}{da}=-\left(r+\eta\right) V=-\xi V$ (A3)

$\frac{dS}{da}=\beta C-\left(\chi+r_{S}\right) S$ (A4) $\frac{dC}{da}=\eta V+\chi S-\left(\beta+\mu^{P}\right) C$ (A5)

$\frac{dU}{da}=rV+r_{S}S+\rho\left(D+W\right)-\left(\delta+\mu^{P}\right) U$ (A6)

$\frac{dD}{da}=\delta U+\mu^{P}P_{D}-\left(\rho+\omega\right) D$ (A7)

$\frac{dW}{da}=\mu^{P}\left(U+P_{W}\right)-\left(\rho+\omega\right) W$ (A8)

$\frac{dP_{D}}{da}=\omega D-\left(\beta+\mu^{P}\right)P_{D}$ (A9)

$\frac{dP_{W}}{da}=\omega W-\left(\beta+\mu^{P}\right)P_{W}$ (A10)

Note that new partnership states are distributed by age of partner.

| **Symbol** | **Label** | **Transition** | **Description** |
| --- | --- | --- | --- |
| *ξ*(*a*) | First sex rate | V → (C,U) | Rate of virgin to sexually experienced. |
| *r*(*a*) | First marriage rate | (V,S) →U | rate of first marriage for never-married |
| *r_S_*(*a*) | First marriage rate for S state | S → U | Rate of marriage for single, sexually-experienced, never-married. *r_S_* = *r* (1+*C*/*S*). |
| *ρ*(*a*) | Remarriage rate | W → U  D → U | Rate of re-marriage for previously-married. |
| *η*(*a*) | Virgin to couple rate | V → C | Rate of virgin to non-marital “coupled” state.  *η*(*a*) = *ξ*(*a*) *− r*(*a*) |
| *χ* | “Couple”  formation rate | S → C | Rate of single, sexually-experienced, never-married, to non-marital “coupled” state. |
| *ω* | “Partnership” formation rate | W → P_W_  D → P_D_ | Rate of single, previously-married (widowed or divorced) to non-marital “partnered” state |
| *β* | Breakup rate | C → S  P_W_ → W  P_D_ → D | Dissolution of non-marital relationships due to separation |
| *δ* | Divorce rate. | U → D | Dissolution of marriages due to divorce |
| *μ^P^*(*a*) | Partner mortality rate | U → W  C → S  P_W_ → W  P_D_ → D | Death rate of partners of individuals in cohort |

**Table A1:** partnership state transition rates

**Marriage market**

We define the following:

*δt* Time-step

*P* Population size

*R* Population union formation rate

$\Delta u$ Total number of new unions during *δt*

$\Delta u_{i}$ Number of females age *i* entering new unions

$\Delta u_{j}$ Number of males age *j* entering new unions

$\Delta u_{ij}$ Number of new unions of females age *i* with males age *j*

$f_{ik}$ Number of susceptible females age *i* in state *k*

$m_{jk}$ Number of susceptible males age *j* in state *k*

$r_{ik}$ Recruitment rate (females age *i*, state *k*)

$r_{jk}$ Recruitment rate (males age *j*, state *k*)

The total number of new unions in the population is given by

$\Delta u=(R\delta t)P$.

For females and males respectively, the numbers of new unions by age are

$\Delta u_{i}=\delta t\sum_{k} r_{ik}f_{ik}$ and $\Delta u_{j}=\delta t\sum_{k} r_{jk}m_{jk}$.

These are marginals of $\Delta u_{ij}$, and their sums over age are equal to $\Delta u$. The probability that a female entering a new union is of age *i*, and a male entering a new union is of age *j*, are:

$p_{i}=\frac{\Delta u_{i}}{\Delta u}$ and $p_{j}=\frac{\Delta u_{j}}{\Delta u}$ .

The probability that a new union is between a female age *i* and a male age *j* may be written as

$p_{ij}=p_{i}p_{j}\varphi_{ij}$,

where$\varphi_{ij}$ is the “affinity matrix”. Hence, the number of new unions in the age cell (*ij*) is

$\Delta u_{ij}=p_{ij}\Delta u=\frac{\Delta u_{i}\Delta u_{j}}{RP\delta t}\varphi_{ij}$.

During the simulation, *P* may vary, as well as Δ*u_i_* and Δ*u_j_* (since $f_{ik}$, $m_{jk}$, and some recruitment rates may vary), but the matrix $\Phi_{ij}=\frac{\varphi_{ij}}{R}$ is constant, and is calculated at initialisation as

$\Phi_{ij}=\frac{\Delta u_{ij}^{0}P_{0}\delta t}{\Delta u_{i}^{0}\Delta u_{j}^{0}}$. (A11)

Then for each time-step in the simulation the number of new unions, distributed by age of both sexes, is given by

$\Delta u_{ij}=\Delta u_{i} \Delta u_{j}\Phi_{ij}\frac{1}{P \delta t}$. (A12)

The initial state of the population is found by solving the partnership state equations numerically, and then applying a fixed-point iteration method to find a distribution $\Delta u_{ij}^{0}$, with marginals $\Delta u_{i}^{0}$ and $\Delta u_{j}^{0}$ for a stable population of size *P*_0_.

**Epidemiological Module**

The epidemiological module computes new HIV infections and HIV staging. New HIV infections are calculated during time period *δt* for HIV-discordant sexual contact. The number of contacts during the time period is $N=\kappa\delta t$, where *κ* is the rate of sexual contacts per time unit. Given infection probability *q* per contact, the infection probability during the time period is

$\pi=1-\left(1-q\right)^{N}$. (A13)

The value of *q* is calculated from a baseline value, and then amplified by a set of risk factors specified in the simulation.

HIV staging is modelled by computing a waiting time *t_wn_* for an individual in stage *n*. Thus, on infection, the first waiting time for the primo-infection stage is computed and recorded. When that waiting time has expired, the individual moves to the next stage, and so on, until the final stage of AIDS has expired, resulting in AIDS death. Each waiting time is computed from a Weibull distribution given by

$z\left(t_{w}\right)=\exp\left[-\ln2 \left(\frac{t_{w}}{t_{m}}\right)^{S}\right]$, (A14)

where *t_m_* is the median waiting time, and *S* is the shape factor, for the specified stage.
