# Supplementary material for: Fitting the HIV Epidemic in Zambia: A Two-Sex Micro-Simulation Model
Source: PLoS One. 2009 May 5;4(5):e5439. doi: 10.1371/journal.pone.0005439 (PMC2673026; doi:10.1371/journal.pone.0005439)
Supplement: Appendix S2 — (0.32 MB DOC) [file pone.0005439.s002.doc]

**Appendix B**

Figure B-1: Age specific fertility rates

Figure B-2: Age specific mortality rates (without HIV/AIDS)

Figure B-3: Results from fitting the Picrate model to proportions ever married and who ever had intercourse

Figure B-4 : Bivariate gamma distribution of age of husband and wife, first marriage

Figure B-5 : Bivariate gamma distribution of age of husband and wife, remarriage

Figure B-6: Bivariate gamma distribution of age of husband and wife, premarital relationship

Figure B-7: Bivariate gamma distribution of age of husband and wife, extra- or post-marital relationship
