# Supplementary figures and images for: Fitting the HIV Epidemic in Zambia: A Two-Sex Micro-Simulation Model
Source: PLoS One. 2009 May 5;4(5):e5439. doi: 10.1371/journal.pone.0005439 (PMC2673026; doi:10.1371/journal.pone.0005439)

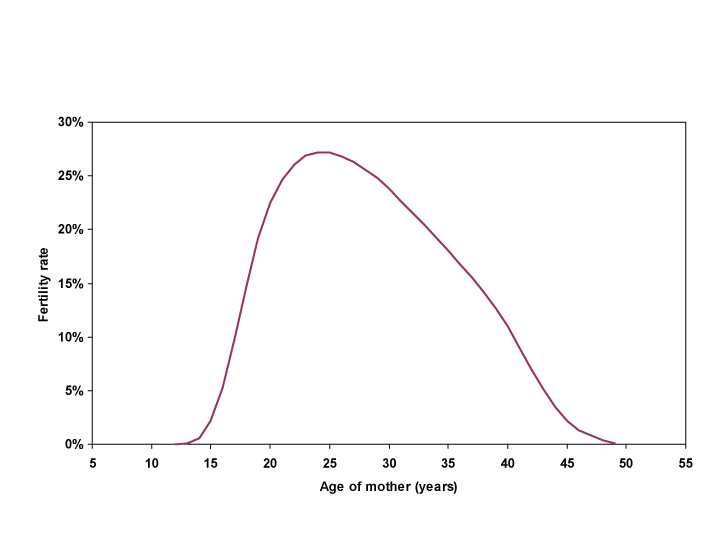

Supplement: Figure S1 — Age pattern of fertility, Zambia. (0.11 MB TIF) [file pone.0005439.s003.tif]

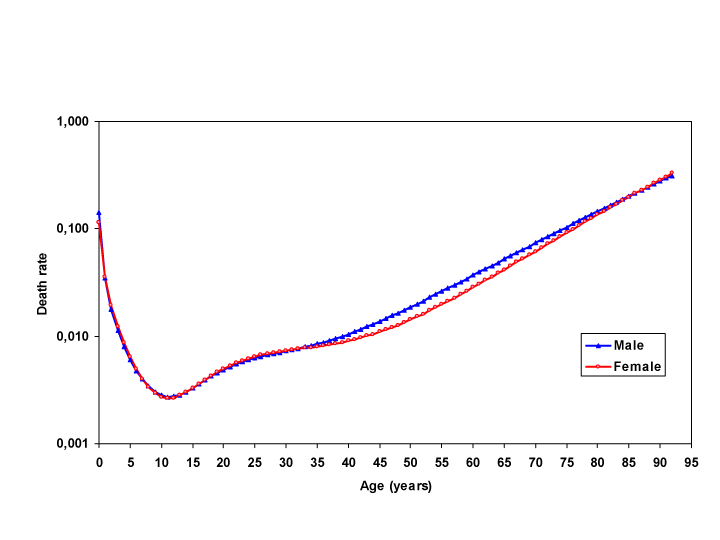

Supplement: Figure S2 — Age pattern of mortality, without HIV/AIDS, Zambia. (0.12 MB TIF) [file pone.0005439.s004.tif]

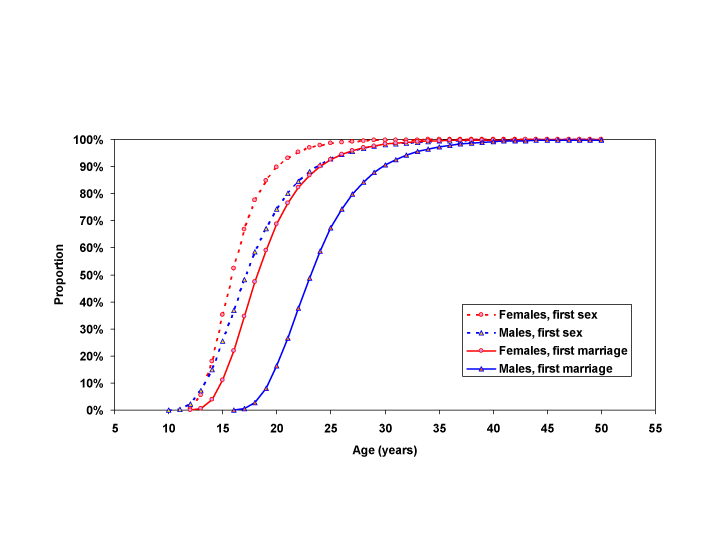

Supplement: Figure S3 — Proportions ever married, and ever had intercourse, after from fitting with the Picrate model. (0.14 MB TIF) [file pone.0005439.s005.tif]

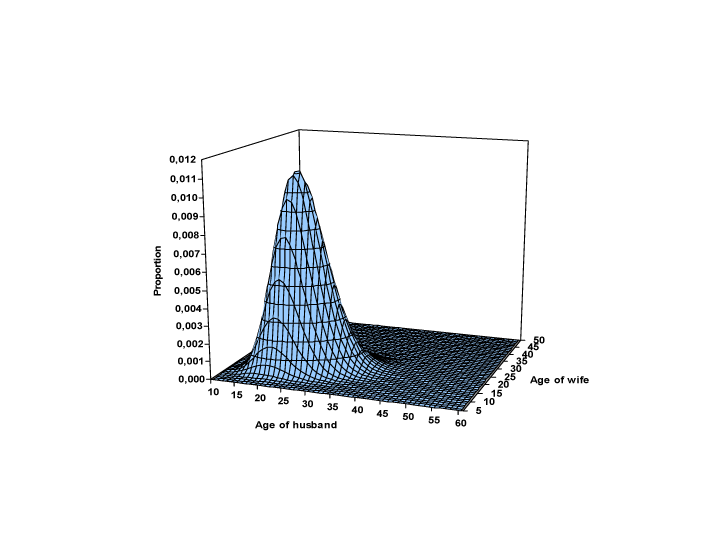

Supplement: Figure S4 — Bivariate gamma distribution of age of husband and wife, first marriage. (0.21 MB TIF) [file pone.0005439.s006.tif]

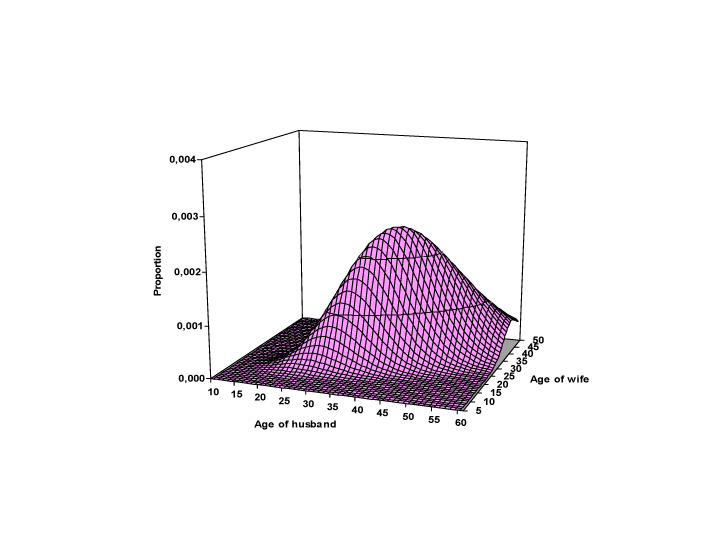

Supplement: Figure S5 — Bivariate gamma distribution of age of husband and wife, remarriage. (0.22 MB TIF) [file pone.0005439.s007.tif]

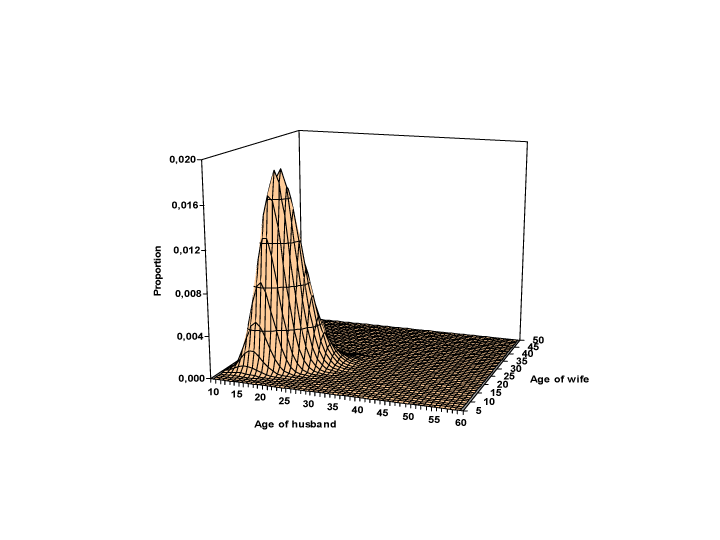

Supplement: Figure S6 — Bivariate gamma distribution of age of partners, premarital relationship. (0.20 MB TIF) [file pone.0005439.s008.tif]

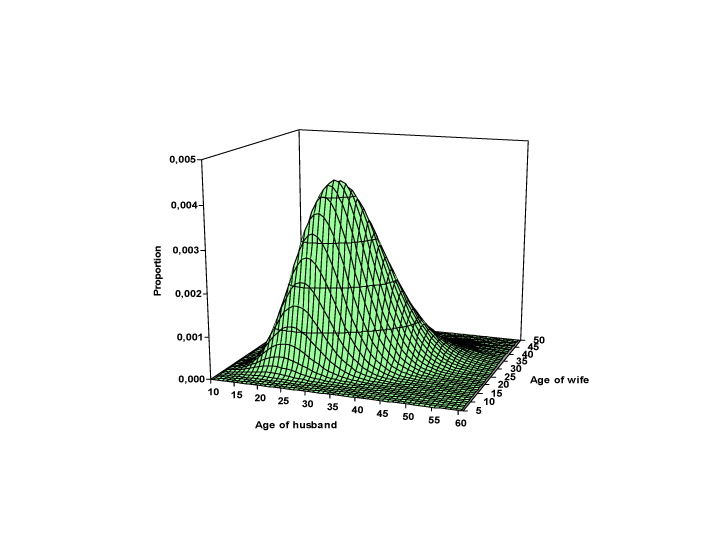

Supplement: Figure S7 — Bivariate gamma distribution of age of partners, extra- or post-marital relationship. (0.22 MB TIF) [file pone.0005439.s009.tif]
